# Reversible cerebral vasoconstriction syndrome following ciltacabtagene autoleucel therapy for relapsed multiple myeloma: a case report

**DOI:** 10.3389/fimmu.2026.1783051

**Published:** 2026-04-02

**Authors:** Tuan L. Phan, Adel Hijazi, Romi Xi, Mohamed Ridha, Tirisham V. Gyang, Monica Sarkar, Mohammad Shujaat, Shahid Nimjee, Andrew Slivka, Mhd Ezzat Zaghlouleh, Allison Jordan, James S. Blachly, Jonathan Brammer, Evandro Bezerra, Naresh Bumma, Don Benson, Elvira Umyarova, Abdullah Khan, Srinivas Devarakonda, Francesca Cottini, Ashley E. Rosko

**Affiliations:** 1 Division of Hematology, Department of Medicine, The Ohio State University Comprehensive Cancer Center, College of Medicine, The Ohio State University Wexner Medical Center, Columbus, OH, United States; 2 Department of Neurology, The Ohio State University, Wexner Medical Center, Columbus, OH, United States; 3 Department of Radiology, The Ohio State University, Wexner Medical Center, Columbus, OH, United States; 4 Department of Neurosurgery, The Ohio State University, Wexner Medical Center, Columbus, OH, United States

**Keywords:** ciltacabtagene autoleucel (cilta-cel), midodrine, multiple myeloma, neurovascular adverse reaction, reversible cerebral vasoconstriction syndrome (RCVS)

## Abstract

Ciltacabtagene autoleucel (cilta-cel), a BCMA-directed CAR-T therapy, is approved for relapsed/refractory multiple myeloma (RRMM). Cytokine release syndrome (CRS) and immune effector cell-associated neurotoxicity syndrome (ICANS) are common, but reversible cerebral vasoconstriction syndrome (RCVS) is a previously unreported complication following cilta-cel. A 63-year-old female with RRMM (IgA lambda, t(14;16)), no vascular risk factors, received cilta-cel after two prior therapies, including autologous transplantation. On day +10 post-CAR-T infusion, she developed grade 1 CRS, managed with tocilizumab and vasopressors, including midodrine. On day +19, she presented with headache, leg weakness, and loss of fine motor skills. Neuroimaging showed multifocal cerebral stenosis and ischemic strokes, initially suggesting vasculitis. Subsequent imaging confirmed RCVS (RCVS2 score 9). Treatment with corticosteroids, anakinra, siltuximab, cyclophosphamide, verapamil, and nimodipine failed to halt neurological decline, resulting in right-sided hemiplegia and Gerstmann’s and Balint’s syndromes. By day +69, CT angiography showed resolved stenosis, but neurological deficits persisted. This case identifies RCVS as a novel, life-threatening complication following cilta-cel, possibly linked to CRS, tocilizumab, or midodrine. It underscores the need for broader differential diagnoses and tailored management for CAR-T neurotoxicities, warranting further research.

## Introduction

Ciltacabtagene autoleucel (cilta-cel), a B-cell maturation antigen (BCMA)-directed chimeric antigen receptor T-cell (CAR-T) therapy, is approved for patients with relapsed or refractory multiple myeloma (MM) who have received at least one prior line of therapy, including a proteasome inhibitor and an immunomodulatory agent, and are refractory to lenalidomide (1). Cilta-cel can offer long-term durable disease control (2) and is associated with known toxicities, primarily cytokine release syndrome (CRS) and immune effector cell-associated neurotoxicity syndrome (ICANS) (3). Cilta-cel is also associated with atypical neurological toxicities, including a disproportionately high rate of CNS hemorrhage and cerebrovascular accidents compared to idecabtagene vicleucel (4). While delayed neurotoxicity events such as movement and neurocognitive treatment-emergent adverse events are being increasingly recognized (e.g. cranial nerve palsies and parkinsonism), neurovascular complications are not well-documented complications of cilta-cel. Reversible cerebral vasoconstriction syndrome (RCVS) involves transient vasospasm and can lead to ischemic stroke, presenting with focal neurological deficits, altered mentation, or vision loss. It is often triggered by vasoactive drugs or postpartum states and is differentiated from vasculitis by its reversibility and lack of inflammatory markers (5). Herein we describe a case of a 63-year-old female with relapsed MM who developed multiple ischemic strokes following cilta-cel infusion attributed to RCVS, a previously unreported complication of CAR-T therapy. This case highlights a previously undescribed and life-threatening neurotoxic complication of CAR-T therapy, underscoring the need for heightened vigilance and further investigation into its pathophysiology and management in patients without traditional risk factors.

## Methods

### BCMA flow cytometry

Approval for specimen procurement was obtained under a protocol approved by the Ohio State University Institutional Review Board. Whole blood samples were collected in EDTA tubes and used for BCMA-CAR-T cell analysis. Each sample was processed using the standard Beckman Coulter TQ-Prep protocol (Beckman Coulter, Cat. No. NC1908934). After treatment, samples were washed with phosphate-buffered saline (PBS) and the TQ-Prep reagent was removed by decanting. For BCMA-CAR staining, the fluorescein isothiocyanate (FITC)-labeled human BCMA protein detection reagent (Acro Biosystems, Cat. No. BCA HF254) was used. An FITC-labeled human fibroblast activation protein (FAP) protein (Acro Biosystems, Cat. No. FAP HF263) served as the isotype control. Samples were incubated at 37 °C for 60 minutes. Following this, CD2-APC-A700 (Beckman Coulter, Cat. No. B12111), CD3-APC-A750 (Beckman Coulter, Cat. No. A66329), CD19 FITC (Beckman Coulter, Cat. No. B36534), and CD45 Krome Orange (Beckman Coulter, Cat. No. A96416) antibodies were added, and samples were incubated in the dark for an additional 15 minutes. After a final PBS wash, samples were acquired on a Beckman Coulter Navios EX flow cytometer. Data were compensated and analyzed post-acquisition. The absolute BCMA-CAR-T cell count was calculated by multiplying the percentage of positive cells by the absolute lymphocyte count.

## Case report

### Pre-transplant clinical course

The patient is a 63-year-old female with no significant past medical history and notably had no history of diabetes, hypertension, cardiovascular, or autoimmune diseases. She was diagnosed with ISS stage 2 IgA lambda multiple myeloma (MM) with high-risk cytogenetics t(14;16) in 2014 and underwent induction with cyclophosphamide, bortezomib, dexamethasone (CyBorD), achieving a very good partial response (VGPR), followed by autologous hematopoietic cell transplantation (autoHCT) with maintenance lenalidomide. After 10 years and 1 additional line of therapy (daratumumab, bortezomib, and dexamethasone [Dara-Vd]) for relapsed MM, she underwent T-cell procurement and bridging treatment with pomalidomide, carfilzomib and dexamethasone (KPd) followed by standard lymphodepletion (LD) with fludarabine and cyclophosphamide. Disease was in a partial response (PR) at time of CAR-T.

### Cellular therapy clinical course

**Figure 1:**
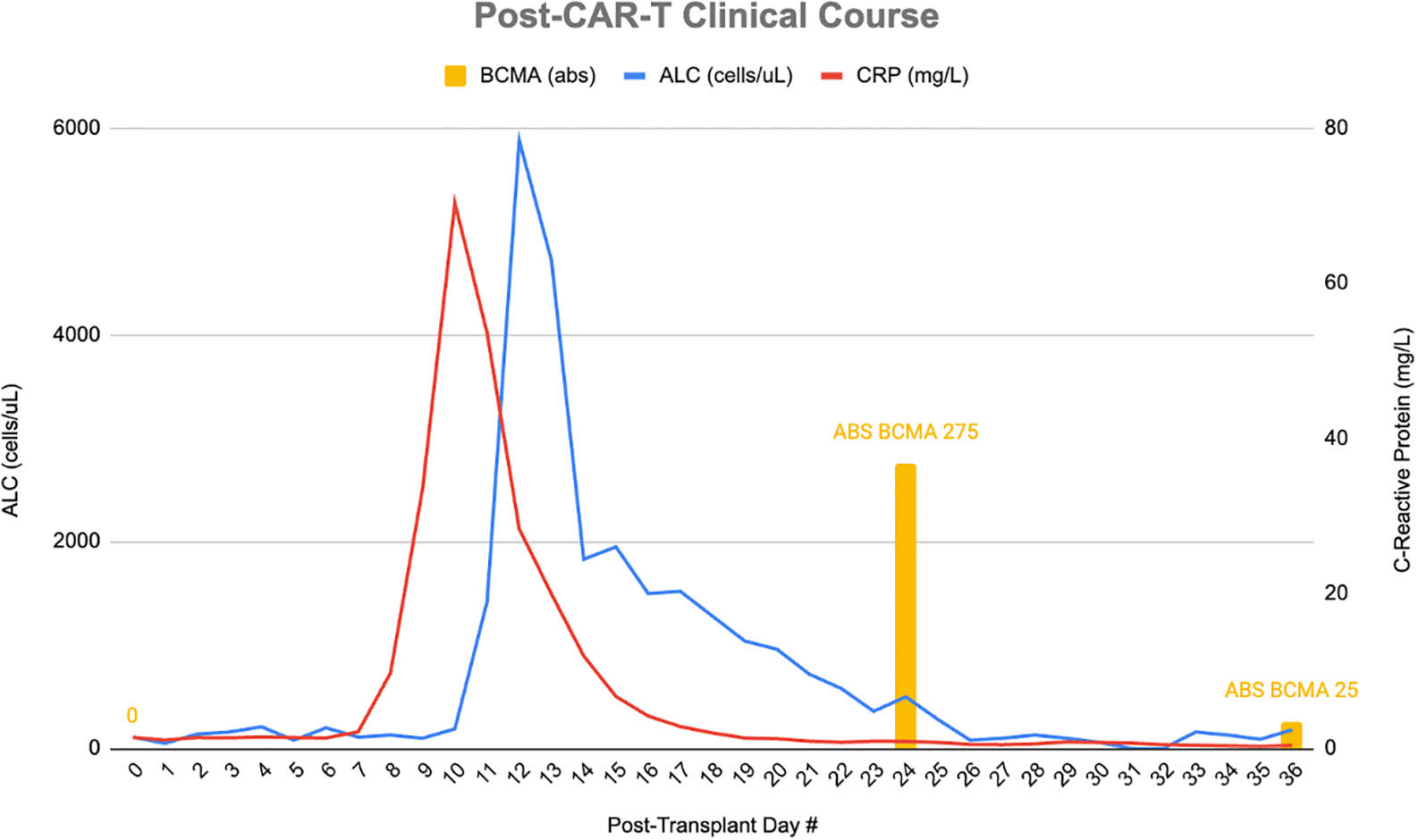
Clinical course showing ALC, CRP, and absolute BCMA counts.

The patient was admitted on CAR-T day -1 with nausea, vomiting, and abdominal pain following LD treatment. On Day 0 she received cilta-cel infusion (**Supplementary Table 1**; **Figure 1**); baseline endothelial activation and stress index (EASIX) (6) and modified EASIX (m-EASIX) (7) scores were 0.82 and 0.20, respectively. On day +10, she developed a fever (101°F) and tocilizumab was initiated for grade 1 CRS, however hypotension ensued, unresponsive to 500mL lactate ringers, 2L normal saline and 25g (5%) albumin bolus, necessitating norepinephrine infusion. Dexamethasone 10 mg every 6 hours combined with a second dose of tocilizumab were administered, and she was transferred to the intensive care unit (ICU). On day +11, she experienced acute left neck and eye pain, which responded to IV hydromorphone and self-resolved initially. Infectious workup was unremarkable and antibiotics were discontinued. On days +12 and +13, high absolute lymphocyte count (5.88K/µL and 4.72 K/µL, respectively) prompted dexamethasone 10 mg twice daily for 3 days to prevent neurotoxicity. Due to persistent orthostatic hypotension, midodrine 2.5mg was started on day +14. On days +14–15, the patient developed recurrent undifferentiated shock and antibiotics were resumed empirically.

**Figure 2:**
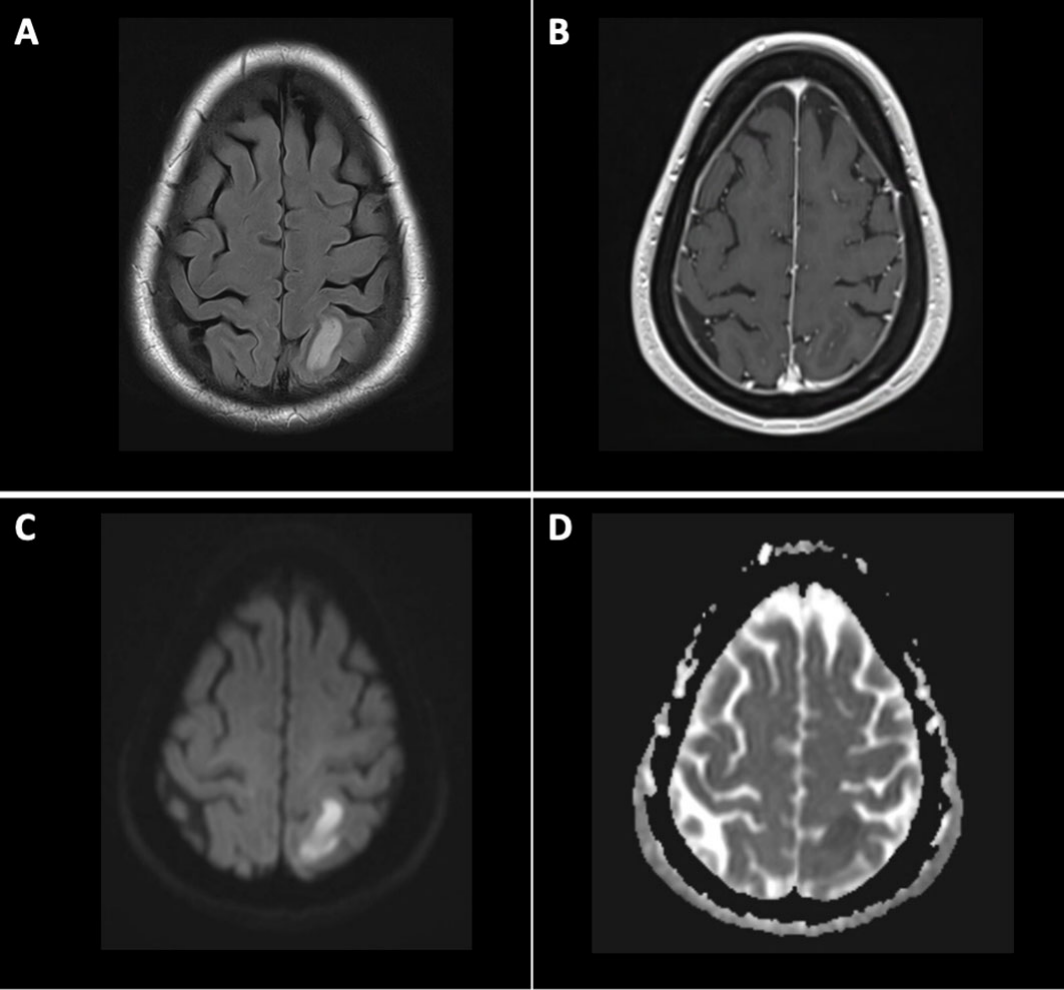
MRI on post CAR-T day +21. MRI Brain with and without contrast showing T2 hyperintensity **(A)**, lack of enhancement on T1 post-contrast **(B)**, diffuse restriction **(C)**, and corresponding ADC correlate **(D)**.

**Figure 3:**
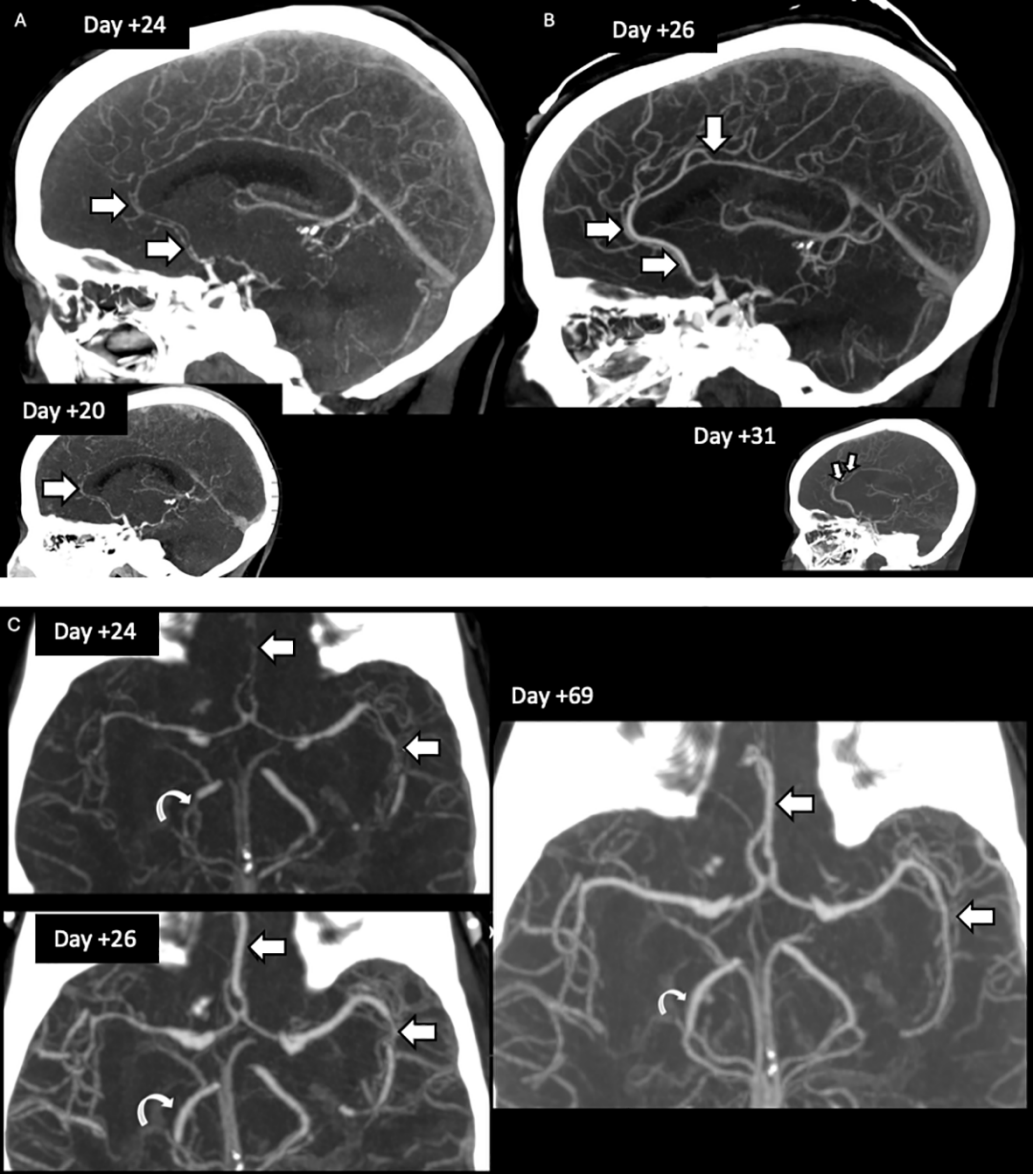
CT angiogram brain/neck. **(A)** Global vascular irregularity with segmental luminal narrowing’s seen in ACAs (short arrow), both MCAs but more evident in the left MCA (long arrow), and right PCA (curved arrow) as seen on initial presentation CTA Brain and Neck (day +20 to +24). **(B)** Follow up CTA Brain and Neck shows waxing-waning of luminal irregularities with improved patency of right PCA (curved arrow) and ACAs (short arrow). **(C)** Post discharge CTA Brain and Neck imaging showing interval resolution of multifocal stenoses in ACAs (short arrow), MCAs (long arrow), and PCAs (curved arrow). ACA, anterior cerebral artery; PCA, posterior cerebral artery; MCA, middle cerebral artery.

**Figure 4:**
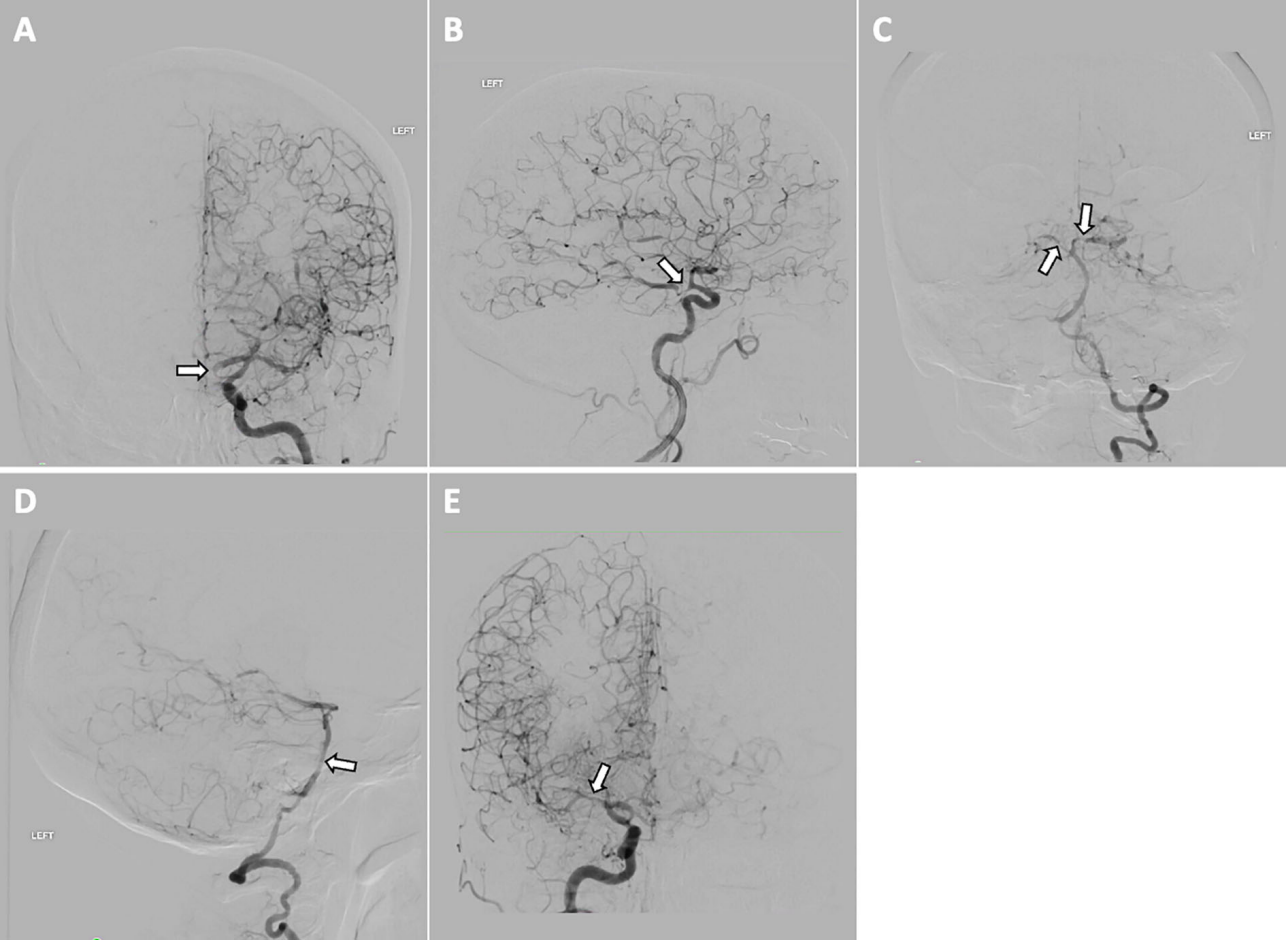
Diagnostic cerebral angiogram showing multifocal stenosis in: **(A)** left middle cerebral artery (MCA; M1) in coronal view, **(B)** left MCA (M1) in sagittal view, **(C)** bilateral posterior cerebral arteries (PCAs; P2s) in coronal view, **(D)** vertebral artery in sagittal view, **(E)** right MCA (M1) in coronal view.

On post-CAR-T day +19, she awoke with inability to move her legs, with weak extremities, and loss of fine motor skills; she remained alert and oriented. Later she had a sudden onset headache behind the eyes with peak intensity occurring in less than one minute after which it lasted for hours. Her symptoms resolved that night, and a computed tomography (CT) head was normal. Neurology consultation was obtained the next day – exam was normal except for non-specific unsteadiness with ambulation and reduced attention. Magnetic resonance imaging (MRI) brain with and without contrast obtained on CAR-T Day +20 suggested possible ischemic stroke versus ICANS (**Figure 2**). CT angiogram (CTA) then showed multifocal, severe stenosis throughout the cerebral vasculature, involving the A2, M2, and P2 branches, along with the cervical vertebral arteries, with areas of beaded appearance suggestive of vasculitis vs. RCVS (**Figure 3A**). Her neurologic symptoms began to fluctuate with right sided weakness and visual complaints until they became persistent on day +22 as a combined Gerstmann’s syndrome (right/left disorientation, finger agnosia, acalculia, and agraphia) and Balint’s syndrome (optic ataxia, oculomotor apraxia, and simultanagnosiais). She was then transferred to the neuro intensive care unit (ICU) and a diagnostic cerebral angiography (DCA) demonstrate a diffuse beaded appearance of the intracranial vasculature with no improvement post-intra-arterial verapamil (**Figure 4**). Two serial lumbar punctures separated by 48 hours showed no detectable nucleated cells, normal protein, at most 32 RBCs, and no infectious etiology. Anti-neutrophil cytoplasmic antibody (ANCA), antinuclear antibody (ANA), and cryoglobulins were negative. Cerebrospinal fluid (CSF) cytokine panel 13 (Arup Laboratories) showed an IL-6 level of 14.2 pg/mL (ref <7.5 pg/mL). Treatment for ICANS was escalated with high-dose IV methylprednisolone, anakinra and siltuximab (11 mg/kg on post-CAR-T day +23). Serum cytokine panel on day +23 (4/17) showed IL-10 level of 11.1 pg/mL (reference range <11.1 pg/mL) and IL-6 level of 2.1 pg/mL (reference range <2.0 pg/mL).

On post-CAR-T day +24, MRI showed worsening strokes with petechial hemorrhage. Absolute lymphocyte count (ALC) on day 24 was 393 cells/uL, 72.3% were BCMA-CAR-T positive (abs 275 cells/µL, **Figure 1**). Progressive neurological decline (inability to name objects, poor handgrip) and worsening MR imaging prompted cyclophosphamide (2g/m²) and IV immunoglobulin (IVIG: 400mg/kg). On days +25–26, she developed right-sided hemiplegia with CTH showing bilateral posterior predominant strokes with hemorrhagic conversion of stroke in distal left ACA territory along with 6–8 mm midline shift. Interval CTA on day+26 showed interval improvement in multifocal stenosis (**Figure 3B**). MRI with vessel wall imaging was obtained on day +28 which showed no wall enhancement. Given the improvement in CTA findings, she was diagnosed with RCVS. Methylprednisolone was tapered (250 mg twice daily to prednisone equivalent) and anakinra was decreased to daily dosing. CT angiography on day +31 showed worsening multifocal vasospasm prompting treatment with nimodipine and later verapamil. The patient had persistence of Balint’s and Gerstmann syndromes (ICE score 6/10 by day +31), with right-sided hemiplegia improved slightly day +32. The patient was given granulocyte colony-stimulating factor (G-CSF) from day +26 to +35 for neutropenia associated with cyclophosphamide administration with appropriate recovery of neutrophils. ALC was 82 on day +36, of which 27.6% were BCMA-CAR-T positive cells (absolute count 25 cells/µL; **Figure 1**). Her ICE scores fluctuated between 7 to 9 (she was unable to write sentences due to stroke sequelae) and she was discharged on day +57 to a stroke rehabilitation center. Final post-discharge CT angiogram of brain/neck on CAR-T Day +69 showed total resolution of multifocal stenosis (**Figure 3C**). Neurological exam on post-CAR-T day +175 follow-up was notable for residual partial right upper quadrant visual field defect and right leg numbness; she was able to ambulate with a wheeled walker and is functionally independent at home. Verapamil was discontinued and aspirin was continued for stroke prevention. She was briefly hospitalized on post-CAR-T +249 to +251 for acute stroke-like symptoms (encephalopathy, aphasia), with imaging showing no new infarct or hemorrhage, and was managed supportively. Post-discharge, she demonstrated marked neurological recovery, returning to baseline with only occasional aphasia and stable chronic deficits. The patient continues to recover with ongoing rehabilitation, and her myeloma remains in remission (VGPR).

## Discussion

This case describes a 63-year-old female with relapsed IgA lambda multiple myeloma (MM) who developed severe cerebral vasculopathy likely due to RCVS, resulting in multiple ischemic strokes following ciltacabtagene autoleucel infusion and cytokine release syndrome (CRS). Workup with MRI brain and CT angiography showed ischemic stroke in several vascular territories with multifocal areas of vascular stenosis. These findings were associated with a rapid expansion in lymphocytes, including CAR-T lymphocytes (8) (**Figure 1**). Digital cerebral angiography (DCA) initially suggested potential CNS vasculitis, however subsequent follow-up imaging showed reversibility of vascular stenosis supporting a diagnosis of RCVS. Treatment with high-dose corticosteroids, anakinra, cyclophosphamide, and IV immunoglobulin failed to halt neurovascular disease progression. This case represents a novel and unreported neurologic complication following CAR-T therapy.

Ciltacabtagene autoleucel is known for neurological complications, primarily ICANS, which typically manifests as confusion, aphasia, or encephalopathy within the first two weeks post-infusion, often in association with CRS. The proposed mechanism for ICANS suggests an immune mediated endothelial activation-induced blood-brain-barrier (BBB) dysfunction, giving rise to CNS inflammation and neurotoxicity (9, 10). Thrombotic microangiopathy with cerebral multifocal hemorrhage has been described in patients with CRS and ICANS. BCMA-targeted CAR-T therapy is also associated with a late-onset neurotoxicity syndrome termed movement and neurocognitive toxicity (MNT or IEC-Parkinsonism) (11–13); this syndrome is distinct from classical ICANS and occurs with a median onset of 36 days (range 14–914 days) after infusion, typically after resolution of CRS and ICANS. Cerebral vasculitis, stroke, and RCVS are not commonly established complications of CAR-T therapy (4). Cerebral vasculitis is often linked to autoimmune diseases, infections, or malignancy-related paraneoplastic syndromes (14), and presents with insidious headache, cognitive decline, or focal deficits, accompanied by cerebrospinal fluid (CSF) inflammation and serologic markers (e.g., ANCA, ANA). This patient’s vasculopathy was atypical: it developed acutely and occurred without predisposing autoimmune or infectious triggers. The patient’s CSF demonstrated borderline elevated IL-6 levels (14.2 pg/mL, ref <7.5 pg/mL) however this was significantly lower than previously reported values (median 60, range 5.5–548 pg/mL) for patients with grade 2–4 ICANS (15). Clinically, the patient’s presentation was also dissimilar from what is expected from RCVS which is recurrent thunderclap headaches while our patient had sudden onset severe headache only early in her course. The absence of response to intra-arterial verapamil and vasoconstriction in the cervical vertebral arteries on imaging argued against RCVS, however repeat vessel imaging showed reversibility of vascular stenosis and MRI vessel wall imaging showed no enhancement. CSF sampling also showed no evidence for inflammation. Taken together these findings are most suggestive of RCVS further supported by an RCVS_2_ score of 9 for thunderclap headache (5), trigger (3), and female sex (1).

There does remain some uncertainty regarding the specific trigger of RCVS. CRS, which occurred on post-CAR-T day +10, may have initiated a systemic inflammatory cascade, amplifying endothelial damage, though the delayed onset of neurological symptoms (day +20) argues against a direct link. Tocilizumab, administered for CRS, has been rarely associated with neurological events in case reports – including RCVS (16). Other drugs, such as midodrine (used for hypotension), are vasoactive and theoretically could contribute to RCVS (17), though this patient was only briefly on midodrine, which has a half-life of only approximately 30 minutes (18). RCVS, considered a spectrum disorder, ranges from benign vasoconstriction to severe forms with ischemic or hemorrhagic complications. Additionally, CAR-T has been associated with posterior reversible encephalopathy syndrome (PRES) (19), a phenomenon related to cerebral autoregulation disruption and breakdown of the blood brain barrier. RCVS and PRES are typically thought to be on a spectrum (20), with RCVS sometimes presenting radiographically very similar to PRES with vasogenic edema involving the parieto-occipital areas. While the imaging findings of PRES are typically reversible; there have been cases of related diffusion restriction causing ischemic strokes and hemorrhage, both intra-parenchymal and subarachnoid (21).

This case underscores the need for broader differential diagnoses in CAR-T-related toxicities. Neurologic sequalae associated with CAR-T primarily address anti-inflammatory pathways (cyclophosphamide, corticosteroids, anakinra, siltuximab) whereas vascular spasm and endothelial dysfunction from CAR-T are targeted differently. Lessons from this case include the importance of considering RCVS as a potential neurologic complication of CAR-T-associated management. Current ICANS management protocols, focused on corticosteroids and anti-IL-6 therapies, may be insufficient for vasculopathic complications; advanced neurologic imaging including diagnostic angiogram and CTAs helped to identify the dynamic cerebral vascular changes in this patient. RCVS should be considered in the differential diagnosis in the evaluation of neurologic changes following CAR-T, particularly when vasoactive agents like midodrine are administered, as management of this entity is distinct from that of ICANS.

The reason for this complication in this patient, without stroke or vascular risk factors, is unknown. High-risk cytogenetics (t(14;16)) and prior therapies may have primed an exaggerated immune response, though the patient did not have markedly elevated biomarkers (e.g., IL-6 or ferritin) to suggest this. It is possible that CAR-T or CRS caused endothelial activation and blood-brain barrier disruption, thus lowering the threshold for RCVS that was ultimately triggered by subsequent tocilizumab or midodrine administration. Unresolved questions include the utility of repeat lumbar punctures or vessel wall imaging and the safety of aggressive immunosuppression in this setting. While the RCVS is the most likely diagnosis in this case, the patient also received treatment for CNS vasculitis (e.g. cyclophosphamide) and it is unclear if this provided benefit. Future research should focus on understanding the pathophysiological mechanism of neurovascular toxicities and identifying predictive biomarkers that will help in establishing guidelines for early intervention. With CAR-T use only increasing, this case highlights the evolving complexity of CAR-T toxicities and the urgent need for enhanced monitoring and novel therapeutic approaches to mitigate life-threatening complications.

## Data Availability

The original contributions presented in the study are included in the article/**Supplementary Material**. Further inquiries can be directed to the corresponding author.
