# Supplementary material for: Reversible cerebral vasoconstriction syndrome following ciltacabtagene autoleucel therapy for relapsed multiple myeloma: a case report
Source: Front Immunol. 2026 Apr 2;17:1783051. doi: 10.3389/fimmu.2026.1783051 (PMC13083160; doi:10.3389/fimmu.2026.1783051)
Supplement: Supplementary file 1 [file Table1.docx]

| SUPPLEMENTAL TABLE 1: POST CAR-T CLINICAL COURSE | | | |
| --- | --- | --- | --- |
| **Post-CAR-T Day #** | **CRS grade**  **ICANS grade**  **EASIX score**  **m-EASIX score** | **Clinical Course/Data** | **Intervention** |
| +10 | CRS 1  ICANS 0 | Fever (101°F) | Tocilizumab #1 |
|  | CRS 3  ICANS 0  EASIX 1.88  m-EASIX 16.36 | Hypotension  Bradycardia | IV fluids and albumin, norepinephrine, cefepime, micafungin  Dexamethasone 10mg q6h (days +10 to +11)  Pt transferred to ICU, tocilizumab #2 given |
| +11 | CRS 0  ICANS 0  EASIX 1.70  m-EASIX 12.95 | Acute left neck/eye pain, self-resolved | IV hydromorphone x1 |
| +13 | CRS 0  ICANS 0  EASIX 1.00  m-EASIX 2.76 | Lymphocytosis ALC 5.88K/uL | Dexamethasone 10mg BID (days +12 to +15) |
| +14 | CRS 0  ICANS 0  EASIX 0.78  m-EASIX 1.54 | Orthostatic hypotension | Midodrine (day +14 to +20) |
| +15 | CRS 0  ICANS 0  EASIX 0.83  m-EASIX 0.83 | Hypotension, lactate 4.3 | Cefepime, IV fluids, albumin |
| +16 | CRS 0  ICANS 0  EASIX 0.72  m-EASIX 0.56 | Random AM cortisol 1.62 mcg/dL | Hydrocortisone 50mg q6h (days +16 to +17) |
| +19 | CRS 0  ICANS 0  EASIX 0.91  m-EASIX 0.23 | Pt woke up unable to move legs, weak extremities w/ loss of fine motor skills, self-resolved  CT head normal |  |
| +20 | CRS 0  ICANS 0  EASIX 0.76  m-EASIX 0.20 | Motor weakness improved but new mild cognitive deficits  MRI brain showed small focal cortical signal abnormality in the left parietal lobe with features of acute infarct in an atypical vascular pattern (Fig 2)  CT angiogram showed multifocal severe stenosis involving the A2, M2 and P2 branches (Fig 3A) | Dexamethasone 4mg q6h (day +20)  Levetiracetam (days +20 to +43) |
| +21 | CRS 0  ICANS 1  EASIX 0.87  m-EASIX 0.15 | ICE 9/10 (unable to write sentence). New right-sided weakness.  CT angiogram showed persistent vasculopathy of cerebral arteries (ACA, MCA, PCA) | Dexamethasone 10mg BID x2 doses  IV methylprednisolone 1g daily (day +21 to +23) |
| +22 | CRS 0  ICANS 4  EASIX 1.37  m-EASIX 0.16 | Combined Gertmann’s syndrome and Balint’s syndrome  Digital cerebral angiography suggestive of global vasculitis given no improvement with intra-arterial verapamil.  Lumbar puncture w/ no evidence of inflammation or infection. | Pt transferred to neurocritical care unit  Nimodipine (x1 day)  Anakinra 100mg BID (day +22 to +25) |
| +23 | CRS 0  ICANS 4  EASIX 1.05  m-EASIX 0.17 | ICE 9/10 (unable to write sentence)  MRI brain showed increase in acute infarcts of posterior cerebral hemispheres, worse on left side. Petechial hemorrhagic transformation noted in left parietal lobe. | Siltuximab 11mg/kg x1  IV methylprednisolone 250mg (day +23 to +27) |
| +24 | CRS 0  ICANS 4  EASIX 0.91  m-EASIX 0.15 | ICE 8/10 (unable to write sentence, mis-identified object)  MRI brain showed evolving acute infarcts in bilateral cerebral hemispheres, worse on left, with increased confluent petechial hemorrhagic transformation, new punctate acute infarcts in the right frontal lobe, and areas of leptomeningeal cortical enhancement.  Transcranial doppler indicated with severe bilateral posterior cerebral artery vasospasm | Cyclophosphamide (2g/m^2^)  IVIG (400mg/kg) |
| +25 to +26 | CRS 0  ICANS 4  EASIX 1.28  m-EASIX 0.20 | Right-sided hemiplegia.  CT head showed bilateral posterior predominant strokes w/ hemorrhagic conversion of stroke in distal left ACA territory with 6-8mm midline shift.  CT angiography brain/neck with improved narrowing in multifocal stenosis (Fig 3B). | Anakinra 100mg daily (day +26 to +30)  G-CSF (day +26 to +35) |
| +28 to +57 | CRS 0  ICANS 4  EASIX 1.58  m-EASIX 0.21 | CT angiography on day +31 showed worsening multifocal vasospasm  ICE score fluctuated between 7-9 out of 10 (unable to write sentence, sequelae of stroke)  Pt discharged to stroke rehab on day +57 | IV methylprednisolone 80mg (days +28 to +30)  64mg (days +31 to +33)  48mg (days +34 to +36)  32mg (days +37 to +39)  16mg (days +40 to +46)  Prednisone 10mg daily from day +47 onwards  Nimodipine (day +34 to day +39)  Verapamil (day +39 to +49) |
